# Lebanese and Syrian refugee parents’ experiences of accessing mental health care for their children in Lebanon: Findings from a qualitative study

**DOI:** 10.1371/journal.pmen.0000305

**Published:** 2025-04-30

**Authors:** Joseph Elias, Bassel Meksassi, Felicity L. Brown, Rozane El Masri, Rayane Ali, Sandy Chaar, Bayard Roberts, Martin McKee, Michele Kosremelli Asmar, Rabih El Chammay, Neha S. Singh

**Affiliations:** 1 Research and Development Department, War Child Alliance, Beirut, Lebanon; 2 Faculty of Public Health and Policy, London School of Hygiene and Tropical Medicine, London, United Kingdom; 3 Higher Institute of Public Health (ISSP), Saint Joseph University of Beirut, Lebanon; 4 Department of Psychiatry, Faculty of Medicine, Saint Joseph University, Beirut, Lebanon; 5 National Mental Health Programme, Ministry of Public Health, Beirut, Lebanon; PLOS: Public Library of Science, UNITED KINGDOM OF GREAT BRITAIN AND NORTHERN IRELAND

## Abstract

Globally, more than 250 million children and adolescents experience mental health (MH) disorders. The estimated 43 million children forcibly displaced at the end of 2022, most commonly displaced to neighbouring low-and-middle-income countries where health and social protection systems are under-resourced, are at especially high risk of developing MH problems. In such settings, host community and refugee parents must often navigate complex pathways to attain the care their children need. Lebanon has experienced multiple crises and now hosts over 1 million refugees from Syria. We explore Lebanese and Syrian refugee parents’ experiences of seeking MH care for their children in Lebanon. This study is part of a larger study qualitatively assessing how Syrian refugee and host populations pay for and access MH services. As part of this study, we conducted a narrative enquiry comprising 10 interviews with purposively selected Lebanese and Syrian parents of children with mental health problems, living in the Greater Beirut and Mount Lebanon area. Data were analysed collaboratively and inductively. Parents reported that the decision to seek MH services for their children commonly followed an acute event, encouragement by family members, or advice from health and child protection organisations. Many described how, even though stigma associated with MH is decreasing in Lebanon, it persists among some communities and creates significant barriers to accessing care, with parents often concealing their care seeking activities. Parents followed a range of pathways to access care, often encountering financial and accessibility barriers. The economic and COVID-19 crises in Lebanon have exacerbated their problems, affecting Lebanese and Syrian families alike. However, Syrian parents needed more support for their children’s basic and educational needs, vital aspects of maintaining their MH and wellbeing. All parents stressed how important it was for MH services to provide comprehensive family support, preserving parents’ dignity, and building trust with both parents and children. They also recommended integrating life skills, recreational activities, and assistance with basic needs into MH services and preferred counselling and psychotherapy over medication. We conclude that Lebanese and Syrian parents in Lebanon face multiple challenges obtaining care for children with MH concerns, a situation exacerbated by the many crises facing Lebanon. Lebanon’s health financing system needs urgent reform to improve MH services to host-community and refugee populations, while governmental and humanitarian stakeholders must seek to deliver cross-sectoral holistic services to children and their families, avoiding the siloed approach that focuses only on children’s MH in isolation from family and social environments.

## Introduction

More than 1 in 10 – or 293 million – children and adolescents worldwide experience mental health (MH) disorders [1], and almost 50% of MH problems have their onset in childhood and adolescence [2]. This age period encompasses about one fourth of the mental disorder burden across the entire life course.[1]However, despite the demonstrated return on investment in effective treatment [3,4], most go untreated, with potentially lifelong consequences. The scale of unmet need is greatest in low-and-middle-income countries, where an estimated 70–90% of those needing treatment lack even minimally adequate care, with children and adolescents particularly neglected [5]. Supply-side factors for this treatment gap include lack of funding [6,7], trained staff, and lack of mental health system capacity and infrastructure, while common demand-side drivers include mental health stigma and inaccessibility of services [8]. The burden is even higher among crisis-affected populations, and that while treatments exist [9–11], the treatment gaps remains extremely higher (>80%) [12].

This enormous disease burden owes much to poverty and associated adversity [13], with an estimated 356 million children worldwide living in poverty in 2017 [14], a figure since increased by COVID-19 [15]. For many, their predicament is exacerbated by armed conflict and other crises; with an estimated 43 million children forcibly displaced by the end of 2022, with the majority in low-and-middle-income countries [16].

These factors come together in Lebanon, a lower-middle income country facing multiple crises including: severe economic problems since 2019 which has led to its currency being devalued by 90%; the COVID-19 pandemic; the Beirut Blast in 2020 – when a warehouse at the Port of Beirut in Lebanon exploded, causing widespread casualties and material damage, and turning the city into a disaster zone; and the consequences of conflict over recent decades, both within its own borders and in neighbouring countries. These crises have further increased unemployment, exacerbated poverty, and reduced accessibility of basic services for both Lebanese and refugee communities, thereby increasing MH needs [17,18]. In March 2023, 80% of the Lebanese population was estimated to be living below the relative poverty line [19]. The situation is especially grave for the over one million refugees in Lebanon, mainly from Syria, most of whom have to borrow to meet basic needs [17]. As a result, approximately 60% of Syrian refugees in Lebanon have reduced their spending on health in comparison to pre-crisis time [17]. In a 2022 survey conducted in host as well as Syrian and Palestinian refugee households, UNICEF reported that almost every 7 out of 10 caregivers in Lebanon described their children as anxious, nervous, or worried, and almost half said they were very sad or feeling depressed every week [20].

The MH care system in Lebanon is fragmented; historically, most MH services were provided by the private sector offering specialised outpatient and inpatient care. In 2014, the National MH Programme (NMHP), which is part of the Ministry of Public Health (MOPH) in Lebanon, was established with the aim of reforming the MH system and scaling up of primary care-level MH services at primary health care centres (PHCs) across Lebanon that are operated by a mix of local and international non-governmental and faith-based organisations [21,22]. The NMHP, in line with the national health strategy [23] and national MH strategy [24,25], has been working with partners – including WHO, UNICEF, UNHCR and non-governmental organisations (NGOs) – to integrate MH services into select PHCs by ensuring the availability of essential psychotropic medications and by providing the World Health Organization’s (WHO’s) MH Gap Action Programme (mhGAP) training and supervision [26] for MH professionals to be able to screen, assess, manage, or refer MH cases when needed [25]. However, this integration has been subject to budget availability as well as to other structural challenges.

Lebanese MH services are also poorly and disparately financed, with MH accounting for only 5% of the overall health budget of the MoPH, most of which is allocated to inpatient care [27]. MH financing arrangements in Lebanon, which largely comprise of humanitarian funding from external sources, are similar to those in other crises-affected settings where funding priorities are decided based on the organisations leading the response, in this case through the Lebanon Crisis Response Plan [18]. Populations face very high out of pocket expenditures, especially since the start of the economic crisis in 2019, with 70% of people requiring support to cover healthcare costs, up from 48% previously [28]. While MH services at PHCs are provided at no or low cost, service users must pay for transportation. Moreover, within the private sector, the majority of private insurance and mutual funds do not cover MH services. The United Nations Refugee Agency (UNHCR) covers 85–90% of fees for specialised and hospital-level MH services for registered Syrian refugees, and various NGOs in civil society attempt to cover Lebanese who cannot afford coverage or insurance.

Parents are often the first to seek help for their children’s MH [29,30] but must often navigate complex pathways to do so [31], a task that has become especially difficult for parents in Lebanon following the economic crisis. Despite the important role of parents in MH care for children, there are only a few studies globally that explore parents’ perspectives on seeking MH care for their children [29,30,32,33], with none from Lebanon or other crises-affected settings. Our study aimed to understand parents’ experiences obtaining MH care for children in Lebanon by both Lebanese citizens and Syrian refugees. This study was conducted as part of GOAL, a multi-partner research project seeking to support health system responsiveness to the MH needs of people affected by protracted displacement in Lebanon [34].

## Methods

### Study design

We conducted qualitative research - specifically employing a narrative enquiry approach [35,36] – by interviewing parents of children who have accessed MH services in Lebanon. Narrative inquiry has gained popularity in health services research and is based on the premise that by listening to the stories of others, we can make sense of their experience and understand how they construct meaning within a broader social context [37]. Specifically, a narrative approach to interviewing places participants at the centre of the study, focusing on their experiences and the meanings they attribute to them [38]. Stories in narrative inquiry are helpful for leading the researcher toward a better understanding of phenomena, which is why this is a useful method for understanding the experiences of parents of children accessing MH services in Lebanon, given their experiences of forced displacement, increasing poverty, socio-economic precarity, political and economic crises [39]. Anderson and Kirkpatrick note the importance of research that centres patient perspectives in improving health services [38]. This study is a secondary analysis of a sub-set of data collected in a broader GOAL study of barriers, facilitators and proposed solutions to equitable mental health financing and service delivery for displaced Syrians and host populations in Lebanon [34]. The broader study interviewed a range of stakeholders including national, United Nations and NGO stakeholders; frontline MH service providers; insurance company representatives; Lebanese and Syrian adults using MH services; and Lebanese and Syrian refugee parents of children aged 12–17 years using MH services. We conducted a sub-analysis of the data from narrative interviews with Lebanese and Syrian parents of children using MH services to allow more in-depth exploration of the issues they faced.

### Study setting

This study was conducted in in the Beirut and Mount Lebanon governorates, which are urban settings including almost half of the country’s population [40]. This region hosts around 29% of the most deprived groups in the Lebanese population, 22% of registered Syrian refugees, 22% of the poorest Palestinian refugees, and 14% of the poorest Palestinian refugees from Syria [40,41].

### Study participants and sampling

Parents of children aged 12–17 years receiving MH services from PHCs and NGOs were recruited through snowball sampling with the support of humanitarian organisations, PHCs, and NGOs providing or involved in the delivery of MH services. We sampled displaced Syrian and Lebanese parents for this narrative inquiry, as they have unique experiences given the multiple crises they have and are continuing to experience. These unique experiences include the war in Syria and being forcibly displaced from there, living in extremely precarious socio-economic circumstances in Lebanon, the political and financial crises in Lebanon, and the Beirut Blast of 2020. We conducted 10 narrative interviews with parents, divided equally between Lebanese and Syrian nationalities. Seven were mothers (4 Lebanese, 3 Syrian) and 3 were fathers (2 Syrian, 1 Lebanese. These interviews were a sub-set of a larger sample (73 interviews and 3 focus group discussions) of the broader study noted above. The small sample size of this study is suitable for a narrative inquiry, which is recommended to be small and purposive because the criteria for selection should include unique, nuanced experiences focused on a lived experience – which is different to if the study had a phenomenological design [35]. We stopped collecting data when saturation was reached, as agreed by the study team after reviewing new data which provided no new themes or ideas [42–44], as part of the collaborative coding approach [45].

### Data collection tools and process

**Fig 1:**
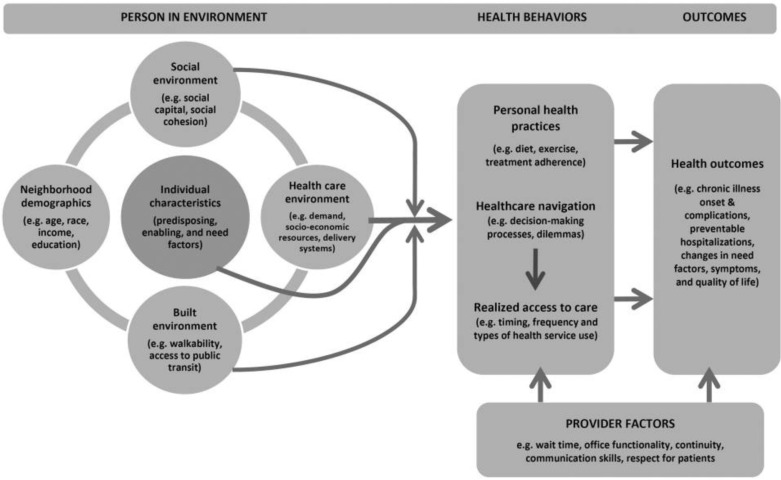
Conceptual framework for examining healthcare access, financing and navigation [46].

Topic guides were developed collaboratively with GOAL project partners with experience working with MH service users in Lebanon. The narrative interview guide had open questions to encourage parents to share their stories and experiences of the reasons for seeking MH care and process of accessing MH services for their children (S1 Appendix). Interview topic guides for the broader study including the narrative interviews were informed by the Ryvicker (2018) conceptual framework (Fig 1), which theorises that healthcare navigation is an ecologically informed process not only because of the spatial distribution of health services and resources (including financing), but because of the spatial distribution of individual and environmental factors that influence individual decision-making, behaviour and available resources (including financing) with respect to service use [46].

Data were collected between November 2022 and April 2023. Interviews were conducted by one male and one female Lebanese researcher (JE, RM) employed by an international nongovernmental organization, with technical and methodological support from a researcher (NSS) employed at a UK-based university. Most interviews were conducted in-person in a location of the participants’ choice, which included offices, homes, and cafes, but some were conducted over Zoom. Interviews were conducted in English or Arabic, depending on the participants’ preference. All interviews were audio-recorded and lasted between 45 and 90 minutes.

### Data analysis

Interviews in Arabic were translated and transcribed verbatim in English by the research team, assisted by an external transcriber. Quality checks were conducted throughout. Data were analysed using an inductive coding approach, based on as themes that emerged in participants’ stories [36,37,43,47]. Specifically, the thematic analysis approach involved finding themes in data by reading through it and identifying patterns in meaning [47]. We coded transcripts in Microsoft Word and extracted themes, codes and related data into a Microsoft Excel spreadsheet.

We followed a collaborative coding approach in order to deepen our understanding of the data, allow space for local interpretations and understandings to emerge, and to adhere to the study’s co-production principles including reciprocity, mutual capacity building, and reflecting on power [45,48]. The collaborative coding process involved two authors (JE, BM) coding the data individually and then meeting bilaterally to review the transcript, discuss their codes and reach consensus. Next, a full team analysis was done in regular group meetings with JE, BM, NSS and FLB to further analyse, define and refine the codes and share perspectives on the data. This was then followed by an individual synthesis, a team meeting, and then writing and feedback [49,50]. As a result, the coding process during the analysis phase was not linear; rather, it followed an iterative and dialogic structure [45,49]. The process of collaborative coding is described in more detail elsewhere [45].

### Ethics

Ethical approval was obtained from Saint Joseph University of Beirut (ref: USJ-2020–224, 19/01/2021), and the London School of Hygiene and Tropical Medicine in London (ref: 22766, 13/01/2021). Participants were provided with an Information Sheet detailing the aim and scope of the study, with written informed consent obtained prior to conducting interviews. All identifying information in the transcripts was anonymised and numerical codes were assigned to each transcript.

## Results

Our inductive approach generated three overarching themes relating to parents’ experiences of accessing MH services for their children: (i) factors influencing parents’ decision to seek MH services for their children; (ii) parents’ experiences of trying to access MH services for their children; and (iii) parents’ experiences with children’s MH services.

### Factors influencing parents’ decision to seek MH services for their children

Parents’ described a number of factors shaping their decision to seek MH services for their children – from environmental (economic crises, COVID-19), societal (shame and stigma related to MH), health care environment (support from MH professionals in seeking care), familial (impact of family dynamics and parenting styles on seeking MH care), and individual (parents’ MH impacting their children; and children themselves asking for help with their MH).

#### Impact of the economic crisis and COVID-19.

Parents described negative changes in their children’s behaviours and MH since the COVID-19 pandemic, often attributing them to lockdown measures and resulting social isolation. However, this was exacerbated by the impact of the economic crisis that accompanied the pandemic, reducing the wages and earning potential of all parents interviewed. In some cases, parents were no longer able to provide for basic needs. The strained economic situation meant that some parents could not secure funds to pay for MH services and, even when services were free, they struggled to secure money for transport.

#### Shame and stigma related to MH.

The shame and stigma associated with MH problems acted as barriers to seeking services for children. While some parents said that that traditional stigma was now less because so many people were affected by the many crises facing Lebanon, thereby serving to normalise seeking help, other parents felt that there is still a long way to go. One Lebanese mother reported: “*Now we are no longer the same. It is not like this [MH] is forbidden*” (CA8). A Lebanese father described taking care about whom he spoke to, given the persistent stigma. He said *“You can’t make the decision on your own [to seek MH care], and you can’t talk about this with anyone, unless they’re professional and specialist, because this can be a topic of shame if you talked to the wrong person”* (CA1). Some parents reported concealing their actions in seeking care from unsupportive partners, family members and friends. One Lebanese mother said: *“I think his [her husband’s] family have some regressive thinking about this topic. They consider the people who receive MH services as sick and not good. As for his friends, I don’t think he shares this topic with them. I don’t think he shares the idea of going to therapy with anyone. He didn’t even start it. He just accepted the idea of getting help.”* (CA4)

Some parents reported feelings of defensiveness if an NGO required their attendance in family therapy sessions. A Lebanese mother explained: *“They [NGO] asked that they wanted to see us together as a family. My husband and children argued against a little at first, but my husband then realised that by doing this step, there is nothing personal against him”* (CA4). Misconceptions and fear about conditions in psychiatric hospitals made parents reluctant to agree to their children being admitted. One Syrian father described how: *“My wife went with us because she wanted to make sure this wasn’t a crazy people hospital, because if it were, she wouldn’t let me admit him [their son]*” (CA10).

In contrast, some parents welcomed growing discussion of MH in schools and communities, which they saw as minimising stigma and encouraging them to talk about their children’s MH problems and seek care for them. One Syrian mother explained how: “*I say yes, the community is accepting the idea of psychotherapy*” (CA3). Some parents also described how their greater awareness of their unhealthy or harmful parenting behaviours and their impact on their children’s MH from feedback from their extended family. This encouraged them to seek help.

Parents also recognised how sharing their own experiences and knowledge about MH with others could help combat stigma while spreading knowledge and encouraging others to seek support when needed.

#### MH care suggested by child protection or health care professionals.

Most parents reported that the decision to seek MH services was prompted by health professionals and child protection personnel. However, they had to overcome concerns about losing their children to court custody – one Lebanese father explained how “*I was just concerned if they wanted to take my daughter away from me*” (CA1). Parents found being approached by child protection organisations and juvenile courts to be a positive factor, as they provided solutions for parenting challenges and child MH issues. One Lebanese mother said: “*This was a beneficial thing to my life. I got to know great people at [NGO]. I was relieved from most of the stress and anger that I had*” (CA2). Another Lebanese mother explained: *“The social worker at [NGO] tried to convince my husband of this step. She convinced him, because he used to always blame others, mainly me. This is his character. She explained that by initiating this step, he would feel better. I felt he accepted this”* (CA4).

Parents who received advice from health professionals reported fewer concerns about the services on offer and the risk that their children might be taken away from them. All parents described how the willingness of health professionals to address their concerns encouraged them to seeking care for their children.

#### Impact of family dynamics and parenting style on children’s MH.

Many parents were aware of the impact of parenting styles on their children’s MH. For instance, a Lebanese father reported how his spouse’s parenting style negatively impacted his daughter’s MH: *“These actions [distressed behaviour by his daughter] are coming from the mother because she is always afraid for her daughter. This has caused our daughter to feel anxious and depressed.”* (CA1). Yet some parents also described how they or their spouses used physical punishment or adopted angry and aggressive reactions when their children misbehave, with consequences for their MH.

The role of family dynamics was also recognised. One Lebanese mother described how her heavy workload and tensions with her husband affected her children: “*I fight a lot with my husband. Probably my long working hours are affecting the house, as the father is more present in the house*” (CA4). In other cases, some parents reported that their relationships with their children impact their child’s behaviour, as a Lebanese mother mentioned: “*She [her daughter] doesn’t set limits [for herself]. She is rebellious, and doesn’t want me to know where she goes*” (CA5).

Given this level of awareness, parents appreciated when MH service providers included their own behaviour and family dynamics in treatment plans, going beyond a narrow focus on the child. For example, a Lebanese mother highlighted the work her family is doing to improve the family environment to reduce their children’s MH issues: “…*We are working together to create the appropriate environment that would help our children. All of us are collaborating to achieve that*” (CA4).

#### Parent’s MH impacting children.

Parents shared insights into the intricate relationship between their own MH and their children’s wellbeing. Several parents described experiencing MH issues themselves, often exacerbated by the ongoing crises, which in turn impacted their children’s MH. For example, the first thought a Syrian father expressed during his interview was about his own MH suffering before describing MH issues his child was facing (CA6). A Lebanese father also highlighted the need for MH support for his wife, emphasising that addressing the MH needs of parents is integral to the overall well-being of the family: “*My wife should receive the help before my daughter*“(CA1).

Parents also highlighted how parental MH impacted family dynamics. As a Lebanese father said: “*You should first address the parents’ [MH], then begin with the child*” (CA1).

#### Children reaching ‘crisis’ point before parents’ seeking care.

Parents faced difficulties deciding when to seek care, often delaying until symptoms were severe, for example when there were suicide attempts or psychosis. One Syrian mother described how severe symptoms were the catalyst for deciding to seek help for her daughter “*I sensed that there is something wrong with my daughter, based on her behaviour. For example, she attempted suicide. She threw herself from the balcony, she cut herself. All these were signs to know that my daughter is not fine*.” (CA9) A Syrian mother shared: “*My daughter cut herself around 3 times. She prefers to sit alone for long periods of time and doesn’t respond when people call her. She is not the same person she was in Syria. That’s why I thought of doing something for her. That’s when we attended the [MH] sessions and thankfully, we, all of us, benefitted from them a lot.” (*CA7).

#### Children asking for parents’ help.

Highlighting the importance of children’s awareness of MH, one Lebanese mother realised the need for seeking help when her child herself asked for support: “*Maybe she hurt herself to get our attention. And she used to act in a weird way at home. I used to ask her, but I wasn’t in a good place to see what’s wrong, but thankfully she asked to get help. I think the reason she asked because she felt the need to be heard, and I wasn’t listening to her, and her father didn’t as well. Maybe this was a statement that ‘I exist’”* (CA4).

### Parents’ experiences of trying to access MH services for their children

Parents reported navigating numerous pathways when trying to access MH services for their children. All parents agreed that the high cost of MH services or costs associated with getting to the MH services (e.g., lost wages, transport) were barriers to accessing MH services for their children, though displaced Syrian parents reported having financial coverage for most of their children’s inpatient MH care. Accessibility of MH services was also reported as a barrier to parents seeking MH care for their children, with long waiting lists, geographical inaccessibility, and limited opening times of MH facilities reported as the main barriers.

#### Parents navigate multiple and ad hoc pathways to access MH services for their children.

Parents reported multiple, and often ad-hoc, pathways through which they enabled their children to access MH services, ranging from use of acquaintances to children’s recreational activities, to NGOs and PHCs, and via the UNHCR for Syrian refugees. Several parents also described the difficulty finding and navigating MH services caused by their lack of knowledge about where and how to find them. A Lebanese father noted: *“As a matter of fact I wanted to seek help before, …but I didn’t know where to go”* (CA1). One Lebanese mother mentioned an element of chance that led her to getting helpful information from an NGO: *“coincidentally, I met someone from [NGO], and they gave me a number to contact, and I did”* (CA4).

Some parents reported paying for treatment at private psychiatric clinics as the waiting lists were so long at the NGO centres. Others were unaware of free or low-cost MH services being provided by NGOs and PHCs. A Lebanese mother who was informed of these services by a client at her shop, but only after she had paid for services in private clinics explained: *“the customer told me there was an organisation that helps in such things. She gave me the number, and I called them*” (CA5).

A Syrian mother described how children’s recreational activities could serve as an entry point for parents navigating services, with children receiving information on MH agencies’ hotlines: “*The primary healthcare centre told me he needed psychological help, so I saw this paper [at a recreational activity centre] and I called. It is a coincidence. Of course, I would’ve reached this place eventually, as I would have asked around*” (CA3).

Syrian refugee parents seeking inpatient care for their children shared similar experiences of being able to get referrals and financial coverage from the UNHCR, which refers cases to NGOs. However, not all Syrian refugee parents were aware that the UNHCR would make these referrals, as revealed by a Syrian father who initially tried to seek care via a private facility: *“When I went to the [private] rehab facility and they asked me for too much money, I called the United Nations, and told them about my situation. They told me they would refer my case to other organizations. I then received several calls from different organizations”* (CA10).

Parents supported by case management or social workers during their therapeutic journey reported a more efficient and pleasant experience than parents who navigated services independently. Child protection organisations often provided a holistic approach, offering family guidance and parenting sessions in addition to MH services, as a Lebanese father reported: *“They told me they would work with the mother and the daughter in order to ameliorate the situation. Because the daughter is depressed.”* (CA1). Parents also reported the benefit of services considering parents’ own MH in addition to their children’s, for instance a Lebanese mother said: “*They [NGO] guided us to the right direction and referred us to another organization that can help me, because I was suffering*” (CA4).

The benefits that parents reported from receiving more holistic care provided by NGOs included greater wellness and provision of housing, supplies, and other basic needs. As a Lebanese mother noted: “*[NGO] even helped me get nutritional goods. They also handed stationary at some point. They also organise activities for the kids. I register the kids there because we can’t afford someplace else. We are poor, but thankfully we are healthy” (*CA2).

#### Financial factors affecting seeking children’s MH care.

All parents identified the high cost of MH services as a barrier to care, especially since the economic crisis. Some Lebanese parents who used private services previously reported being unable to keep up with soaring costs, instead seeking low-cost or free services at PHCs and NGO centres even though they often have long waiting lists: “*I didn’t seek help at first because it’s costly. If it was cheaper, I would’ve gone through it. It would cost a lot if you wanted to do this on your own [through a private clinic]*.” (CA1). With PHCs and NGOs under strain and offering limited services, they sometimes ask parents to pay for tests at private clinics and laboratories for their children, which are often not possible due to the high cost: “*They [NGO] also ordered me to do some tests for her, as she has some growth issues [in addition to MH issues]. I managed to do most of the tests, except one that costs around $400 USD, so I stopped this process*” (CA1).

In contrast, Syrian refugee parents reported full financial coverage of inpatient MH services for their children from UNHCR: “*The UN covers 75%, and this organization covers 15%. And there is another organization covers 10%. So, everything is covered*” (CA10). However, Syrian parents reported facing long waiting lists for inpatient care, sometimes taking up to 6 weeks to get admitted in one case (CA10).

The financial barriers exacerbated by the economic crises in Lebanon were seen as a cause of shame amongst parents, acting as a barrier to care, and to their ability to visit children in hospital. Syrian parents, in particular, described the shame of being unable to meet their children’s basic needs or recreation. A father said: *“I can’t afford to send my kids to football events and trainings. I can’t afford to make my kids happy.”* (CA6)

The high cost of fuel and transport was another barrier facing Lebanese and Syrian parents whose children had repeated visits to MH services. While parents receive some transportation costs from NGOs and PHCs once they reach them, some parents described their inability to find the money up front. As a Syrian father explained: *“I was close to missing this interview, because I don’t have money. I then called someone and they lent me money.”* (CA6). Another Syrian father reported not having enough money to take transport to visit his child in a hospital, even though the hospital would have reimbursed the transport cost: *“I still have an A/C [air conditioner] I am trying to sell, so I can go and see my son. I told the one in the hospital that I may not be able to come because I couldn’t afford it. She told me they would cover the transportation when you come here. I told her I know you would, but I don’t have any money to reach you” (*CA10).

#### Accessibility of MH services.

Parents reported several barriers to accessing services, including long waiting lists, geographical inaccessibility, as well as inflexible opening times of facilities. Parents reported long waiting lists at PHCs, NGO facilities, and hospitals. For example, one Syrian father reported waiting for more than a month to receive follow up calls from organisations providing child MH care: *“I started contacting them, and they told me the doctor is abroad, and he should be the one to admit him. There was a month between the first time I called, and when they contacted me again”* (CA10). In turn, some parents reported being forced to keep trying to get their children seen in private clinics, sometimes calling for support from religious leaders.

Parents who contacted child protection organisations through hotlines reported facing many weeks of inter-agency referrals before being able to get their children the services they needed: “*They just listened at first, and hung up. I called them a few more times, around 2 or 3 times, until they gave me an appointment. They told me they would give me an appointment and they will call me back. I called them every 2 weeks as I remember, I just ignored it, and then when my daughter did something wrong, I called them again. And since then, I’ve been coming here for 2 years now”* (CA5).

Opening hours of facilities often clashed with parent’s working hours, forcing them to make difficult choices to attend the sessions and lose their daily wage or miss appointments. One Syrian father described how: “*They used to contact me to have some [parenting] sessions, but I can’t because every day I am absent, I lose money. And I am a daily worker, so each day I’m not at work, I lose the ability to bring food to the house*” (CA6). Other parents reported prioritising their children’s sessions over work, though this comes at a cost to their daily wages. A Lebanese mother said: *“Sometimes it’s paid, sometimes it’s not. I get one day off, in addition to Sunday. Sometimes I am able to have it on Tuesday, but sometimes I can’t, so I take it as a day off at my expense. But I don’t want to skip any session with [NGO]. I work for my family. It’s ok to lose something for my family”* (CA4)

Although remote services online or by phone were reported as being more accessible, parents reported a preference for face-to-face services: *“Face to face is more important. From my experience… Having online sessions or over the phone is the only solution, as some people don’t have a car. But the result is not the same”* (CA4). Other parents refused remote MH consultations and demanded a face-to-face consultation, as one mother explained: *“The last psychiatrist who told me he would charge 600,000LL [approximately $6.50 USD] and he would conduct the session over the phone, because of COVID, and COVID was nearly over, and we should transfer him the money. I didn’t accept that, and demanded that he sees [my daughter] face-to-face.”*

### Parents’ experiences with and expectations of children’s MH services

Parents reported preferences for MH care for their children, including face-to-face sessions and shifting away from medication-focused treatment to more holistic MH care. As part of this holistic approach, parents emphasised the importance for recreational activities for their children as well as support with school enrolment and for meeting basic needs. They also highlighted the importance of trusting the MH care setting and provider, and how improvements in their children’s MH encouraged them to continue seeking care.

#### Parents’ expectations for holistic MH services.

Many parents expressed a preference for holistic care and a reluctance for their children to “*get used to medications*” (CA5). They described how children, sometimes as young as 10 years old, were given medication rather than attempting alternative approaches such as counselling, with the medicines perceived as achieving minimal or no improvement in their children. Some parents expressed concern that children whose problems were acute, with suicide attempts or self-harm, were repeatedly offered medication without any offer of psychotherapy or other treatments. Most parents rejected this emphasis on medication, opting for alternatives such as improving the family environment and psychotherapy. For instance, a Lebanese mother said, “*The psychiatrist said she should get some medication, but I refused, because I thought there was no need for medications, as we were working together to create the appropriate environment that would help our children*.” (CA4). They also advocated for counselling and psychotherapy approaches as initial treatment for their children, instead of medication being the primary intervention. For example, a Lebanese mother explained: “*The psychiatrist shouldn’t have prescribed medications to individuals as young as my daughter. They should have referred her to a specialist that she just talks to. Here at [NGO], they don’t prescribe medications, they just speak to her, and medications are the last option*” (CA5).

Parents preferred face-to-face sessions rather than remote therapy. They also wanted MH services to include life skills and recreational activities as ways to convey practical messages for their children. For instance, a Lebanese mother suggested that such messages and skills would help children to better deal with challenges they would encounter (CA5). Parents, particularly those struggling financially, also emphasised the importance of providing support for basic needs of children alongside MH services.

Parents saw any shift from medical treatment to counselling and psychotherapy services as an improvement. One Lebanese mother expressed her relief when her daughter no longer required medical interventions: *“She is being treated by words… I was relieved, because I didn’t want her to get used to medications. She was feeling better by talking alone, so this is very good.”* (CA5).

Syrian parents linked their child’s MH issues to the socioeconomic and educational challenges they were facing. These parents expected MH services to be more holistic, and they looked to child protection organisations for help with school enrolment and meeting their children’s basic needs (CA3). Another Syrian parent argued that MH services need to consider the social determinants of poor mental health, including violations of human rights: *“I say why bother giving sessions to kids who work, because they are beaten, and the sessions won’t help them. The UN should help and support them”* (CA3).

Beyond reduction of ‘symptoms’, some parents also expected treatment and counselling to help their children gain self-confidence and acquire life skills. For instance, a Syrian mother said “*I expected that my son gains a bit more self-confidence so he can stand up for himself. And this worked somehow*” (CA3).

#### Trusting and supportive relationship with MH care providers.

Parents and their children reported being encouraged to persist with care where they experienced trust, empathy, respect, and encouragement by service providers. For example, a Lebanese mother described how her interactions with service providers and the guidance they offered alongside the clear treatment plan for her child as inspiring trust: *“They gained my trust fast after a few sessions, because I saw the effect of their guidance. Maybe the effect isn’t apparent yet, but the trajectory is apparent. That made me trust them*” (CA4). A Lebanese mother further noted: “*I felt comfortable here. They [service providers] support you in every aspect, even if they just cheer for you*” (CA2).

Another Lebanese mother also noted the importance of her child receiving MH services in a trusted setting such as a primary health clinic (PHC) where her family has been receiving health care for years: *“Also because here for us in the area this is the [primary health care] centre where we used to go to since we were little … We used to come to this PHC, and I remember whenever we get sick. This centre has been here for us over the years”* (CA8).

Parents were sensitive to perceived slights to their dignity, even when well-intentioned. For example, a Lebanese father perceived a service provider providing a taxi pick up for his child as an act of charity and an affront to his dignity when he was unable to pick them up due to his work schedule: *“I skipped [picking up my child] a few times, so she [service provider] thought I was being reckless. But I had work. She told me I can send them [his wife and daughter] by a cab, and they would cover the expenses. I thought of this gesture as charity. And I don’t like that. I don’t like to send them by a cab”* (CA1).

#### Changes in children’s MH influence parents’ motivation to continue to seek care.

Noticing positive changes in their children motivated parents to continue seeking MH services for their children as well as to initiate help-seeking for other family members, (CA1, CA7). One Lebanese mother said: “*But I can tell from my husband, as he was against this idea [of their child receiving MH services]. However, when he saw it was beneficial, he was convinced*” (CA4).

However, when parents did not notice rapid improvements in their child’s MH, they reported not committing to the prescribed treatment, and their actions ranged between changing service providers to changing treatment approaches.

## Discussion

This is the first study we are aware of to explore Lebanese and Syrian refugee parents’ experiences of seeking MH care for their children in Lebanon. Parents reported deciding to seek MH services for their children based on advice from health and child protection organisations, and in some cases their family members or when their child experienced an acute event such as an attempted suicide. While stigma related to MH issues is reportedly decreasing in Lebanon, it persists in some communities and poses significant barriers to parents seeking MH care for their children, who often end up hiding their decision to do so. Furthermore, parents’ experiences with trying to get their children MH treatment once they have made the decision to seek care so involved diverse pathways, as well as financial and accessibility challenges. The economic crisis and COVID-19 crises have compounded these challenges, impacting both Lebanese and Syrian families alike [51]. However, Syrian parents reported needing more support with meeting their children’s basic and educational needs as a foundation for improving their MH and wellbeing, which is similar to findings from previous studies [52–54]. All parents stressed the importance of MH services providing comprehensive family support, preserving parents’ dignity, and building trust with parents and children alike. They also recommended integrating life skills, entertainment activities, and assistance with basic needs into MH services while preferring counselling and psychotherapy approaches over medication.

Our findings underscore the importance of raising awareness in Lebanon around children’s MH concerns, including reducing stigma, reducing perceptions of blame on parents, where to seek help, and education about what services involve. Several parents reported their children reaching acute psychosis or attempting suicide before they realised the importance of seeking MH care and experiencing long waiting times to access more specialised services. These findings are consistent with a recent study in Jordan which found high rates of suicidal ideation among Syrian refugee children [55], and a a review that found a lack of stigma reduction interventions for children and adolescents in low- and middle-income countries, and calls for more work in this area [56]. There is a need for awareness raising around the early symptoms of MH distress which requires parents’ attention to seek targeted prevention or early intervention services. Addressing stigma and raising awareness in Lebanon could be done by creating a parents of children’s service-users association, drawing on the models of Justice for MH, the first MH service user-led association in Lebanon [57], as well as the Autism Awareness Association Lebanon, which was founded by parents [58].

Parents reported several largely ad hoc pathways through which they decided to seek care or were able to find MH services, which, in addition to the need for awareness raising, also signals the need for improved information and referral systems to help parents seek MH care efficiently. The NMHP has recently launched a website with a list of free or low-cost MH services, including for children [59]. While this website is being disseminated via national platforms, tailored strategies are needed to better publicise it to parents and adolescents. The website should also be made available in Arabic to make it more user-friendly.

Child protection and health providers were reported as a common entry point for addressing both parents’ and child MH, signalling the importance of staff working in these services being equipped with skills in identification and referral, as well as basic psychosocial support. This work is already being done by NMHP in line with the national health strategy [23] and national MH strategy [24,25], via the mhGAP training in the health sector that has been tailored to the Lebanese context and made more comprehensive. This training takes into account integration of MH with other packages of health care at PHC level, as well as human resources. This training includes components for health care professionals to be able to screen, assess, manage, or refer MH cases when needed [25,60,61]. Based on our study findings, this training should also: emphasise professionals providing clear information to parents about what MH services will entail; ensure that parents’ do not feel any blame or shame for needing to seek these services for their children; and acknowledge the need to address other non-MH needs as well. This contextualised mhGAP training should be further scaled up by the MOPH and NMHP, as it is currently provided inconsistently subject to budget availability [62]. Furthermore, as case management services reportedly eased parents’ experiences when navigating MH services, providing them with clear guidance and responding holistically to their concerns, these services should also be further invested in and scaled up in Lebanon. Furthermore, making MH care for both children and parents more accessible and affordable requires comprehensive and inclusive mental health policies and legislations that lead to improvements in MH services, as well as sustainable and culturally adapted MH awareness programmes, including in communities, schools and work settings, that are co-created with target populations.

Our study findings also highlight the importance of delivering MH services to children and their families in a dignified way, while also ensuring accessibility in terms of timing of sessions around caregiver and child needs, location of services, and transportation. Given evidence on home visiting approaches in meeting these needs for children and their families in other settings [63–65], we recommend that these interventions be trialled for children and families who face particular challenges in accessing MH services in Lebanon. Moreover, to ensure responsiveness to the needs of the diverse populations in Lebanon it is essential that MH services, which often draw on Western conceptualisations of mental disorders and treatment, more adequately address service-users’ own explanations for distress and preferences for treatment, including acknowledgement of social justice concerns, stark challenges in meeting basic needs, and involvement of family. This is in line with recent calls for more systematic and careful cultural and contextual adaptations [66], more adequate consideration of social and structural determinants of MH in MH care [67], and improvements in person-centred care [68].

Parents resoundingly stressed the importance of holistic care for families in our study, as parents play an integral role in child MH, and are themselves affected by adversity. Accordingly, there is a need to provide families with support to access all families’ needs, rather than by narrowly focusing MH services on only the child. We recognise the challenge in doing this in a crisis-affected context such as Lebanon, as fragmented MH service delivery in humanitarian settings is often linked to siloed approaches and lack of intersectoral funding, collaboration, and services [69]. Nevertheless, there is a need for humanitarian, development and governmental stakeholders to work together, and across sectors, to implement holistic MH services which could include case management support and family-system level interventions, which have demonstrated feasibility in the region [70]. It would be useful if these recommendations are integrated into the national sub-strategy for child and adolescent MH, which is currently in development by the NMHP in collaboration with UNICEF, WHO and relevant MH actors. It will also be key to meaningfully work with and learn from initiatives such as The United Nations Relief and Works Agency for Palestine Refugees in the Near East’s (UNRWA) Family Health Team approach in Palestine, which takes a holistic and intersectoral approach in meeting children’s and their families’ needs [71].

Our study also found some differences between Syrian and Lebanese parents’ experiences of accessing MH care for their children, though both populations are struggling to access MH services, especially due to the economic crisis in Lebanon. More Syrian parents in our study highlighted their struggles to meet basic and educational needs for their children and asked for MH services to take these needs into account. This finding is consistent with a nationally representative survey of Syrian refugees in Lebanon in 2022 which found that 90% of families needed support to meet their basic survival needs [17]. However, given the compounding crises in Lebanon, the socioeconomic gap between Syrian refugee and Lebanese populations has narrowed, with 80% of the Lebanese population estimated to be living below the relative poverty line in March 2023 [19].

Our study shows disparities in financial coverage for MH services between Lebanese and Syrian populations. Registered Syrian refugees receive coverage through UNHCR, while coverage for Lebanese families depends on insurance or NGO funding. Nevertheless, given that the registration of Syrian refugees by UNHCR in Lebanon was suspended by the government in 2015, there are likely a substantial number of unregistered Syrian refugees facing the same financial barriers to accessing health care as the Lebanese population. Addressing this is important as economic stress is a key social determinant of mental ill health, and can impact on children through poor parental MH and disrupted parenting [52–54]. Financial protection for both populations must be prioritised by governmental and humanitarian actors, which could be achieved by exploring innovative financing mechanisms [24,72].

### Limitations

Our study has several limitations. We only interviewed 10 parents, though we were able to collect rich data using a narrative interviewing approach, which recommends a small and purposive sample size as the criteria for selection should include unique, nuanced experiences focused on a lived experience. We only interviewed displaced Syrian and Lebanese parents who had successfully accessed MH care for their children. Future research should study experiences of parents who are not able to access MH care for their children, to help identify further barriers. Our study was also limited to the Greater Beirut and Mount Lebanon region. Future research should include experiences of parents living in other parts of Lebanon, where services available may be different.

## Conclusions

Lebanese and Syrian parents in Lebanon face multiple challenges in seeking MH care for their children. These challenges have been further compounded by the economic and other crises in Lebanon, which have further pushed both populations into poverty, which impacts their ability to seek timely and quality MH care for their children. Urgent reforms are needed to Lebanon’s MH financing system to equitably and finance MH services for both Lebanese and Syrian refugee populations, aligned with the national health strategy. Governmental and humanitarian stakeholders must also work across sectors to finance and provide holistic services to children and their families, avoiding a siloed approach that only focuses on the MH of children in isolation from family and social environments.

## Supporting information

S1 Appendix Interview Topic Guides. (PDF)
