# Supplementary material for: Lebanese and Syrian refugee parents’ experiences of accessing mental health care for their children in Lebanon: Findings from a qualitative study
Source: PLOS Ment Health. 2025 Apr 30;2(4):e0000305. doi: 10.1371/journal.pmen.0000305 (PMC12798257; doi:10.1371/journal.pmen.0000305)
Supplement: S1 Appendix — (PDF) [file pmen.0000305.s001.pdf]

Interview Topic Guides

English Version

Title of Project: *An assessment of barriers and facilitators to Lebanese and displaced Syrians paying for and accessing mental health services in Lebanon*

Narrative interviews with *parents of children aged 12-17 years* (Lebanese & displaced Syrians)

Main question: Please tell me about your experience of your child using mental health services, starting with why you decided you would seek these services

Probes/ additional questions to ask **ONLY** when participant stops speaking and if not covered in the participant's story/response:

- How did you decide that you were going to seek mental health services for your child? Was your family involved in the decision, or giving advice? Were friends or community members involved in the decision, or giving advice? Did another professional offer advice?
- How did you find out about which services could be available? How did you make your decision about which one to access?
    - Probe for community-based vs. facility-based mental health services
- What were your hopes for the service you were going to access?
- Did you have any doubts or concerns about accessing the service?
- What caused you to overcome those doubts?
- Tell me about the process of accessing the service- e.g. Did you need a referral? did you have to call to make an appointment? who did you speak to? What did they tell you about fees? What information did they give you about the service? How did you feel at each step?
    - Probe: Did you have to pay for these services? If yes, how did they (i.e did it lead to major impact on household budget, borrowing from family, etc.)? If they did not meet the costs, what happened next?
    - *For displaced Syrians only:* Do you receive any vouchers or assistance from any agencies for your child accessing mental health care? If yes, what assistance did you receive?
- In your first appointment, w hat was explained to you about ongoing sessions? How did you feel? did you have any concerns?
- Did your child return to the service? if not, why?
- Has your child attended other mental health services? how do they compare?
- Has your child ever attended once or twice but discontinued? if so, why?
- Had your child ever been advised to seek mental health care in the past, but not done so? if so, why?

- If we wanted to improve the process of accessing services for other children, what would be needed?
    - Probe for improvements in who provides mental healthcare to children; how mental health services for children should be organised and paid for

→ Throughout, ask what could have made this easier for you, or made you feel more confident in accessing the service?
